# Recurrent *PALB2* mutations and the risk of cancers of bladder or kidney in Polish population

**DOI:** 10.1186/s13053-020-00161-y

**Published:** 2021-01-08

**Authors:** Elżbieta Złowocka-Perłowska, Tadeusz Dębniak, Marcin Słojewski, Artur Lemiński, Michał Soczawa, Thierry van de Wetering, Joanna Trubicka, Wojciech Kluźniak, Dominika Wokołorczyk, Cezary Cybulski, Jan Lubiński

**Affiliations:** 1 Department of Genetics and Pathology, International Hereditary Cancer Center, Pomeranian Medical University in Szczecin, Unii Lubelskiej 1, 71-252 Szczecin, Poland; 2 Departament of Urology and Urological Oncology, Pomeranian Medical University in Szczecin, Szczecin, Poland; 3 Gdański Uniwerytet Medyczny Zakład Medycznej Diagnostyki Laboratoryjnej w Gdańsku, Gdańsk, Poland; 4 Department of Pathology, The Children’s Memorial Health Institute, Warsaw, Poland

**Keywords:** 509_510delGA, 172_175delTTGT mutation, *PALB2*, Survival

## Abstract

**Introduction:**

The role of *PALB2* in carcinogenesis remains to be clarified. Our main goal was to determine the prevalence of *PALB2* (509_510delGA and 172_175delTTGT) mutations in bladder and kidney cancer patients from Polish population.

**Materials and methods:**

1413 patients with bladder and 810 cases with kidney cancer and 4702 controls were genotyped for two *PALB2* variants: 509_510delGA and 172_175delTTGT.

**Results:**

Two mutations of *PALB2* gene were detected in 5 of 1413 (0.35%) unselected bladder cases and in 10 of 4702 controls (odds ratio [OR], 1.7; 95% CI 0.56–4.88; *p* = 0.52). Among 810 unselected kidney cancer cases two *PALB2* mutations were reported in two patients (0,24%) (odds ratio [OR], (OR = 1.2; 95% CI 0.25–5.13; *p* = 0.84). In cases with mutations in *PALB2* gene cancer family history was negative.

**Conclusion:**

We found no difference in the prevalence of recurrent *PALB2* mutations between cases and healthy controls. The mutations in *PALB2* gene seem not to play a major role in bladder and kidney cancer development in Polish patients.

## Introduction

Carcinogenesis is an intricate multi-step process initiated by abnormal oncogenic signals in different signaling pathways. Defects in DNA repair are responsible for many cancers like: bladder, prostate, breast, kidney, colorectal, pancreatic, ovarian. The mismatch repair (MMR) and homologous recombination (HR) are well-established DNA repair pathways with links to human cancer [1–7]. In the homologous recombination DNA damage repair a key role play the genes *BRCA1* and *BRCA2* which interacts with many proteins: proteins of the MNR complex (MRE11/RAD50/ NBS1), RAD51, CtIP, MRE11, ATM, H2AX, PALB2, RPA, RAD52 and the Fanconi anemia proteins [8]. Mutations in genes: *PALB2*, *ATM*, *RAD50*, *MRE11*, *NBN* and the genes for the MRN complex are responsible for hereditary cancers. *PALB2* has a large number of interactions with DNA damage response proteins BRCA1, BRCA2, RAD51, RAD51C and XRCC3 which play function in DNA repair by homologous recombination [9, 10]. *PALB2* is not only partner and localizer of *BRCA2* but also is localized and interacts with *BRCA1* plays an important role as a pivotal tumor suppressor protein [11]. Mono-allelic *PALB2* germline mutations disrupt the interaction of *PALB2* with either *BRCA1* or *BRCA2* engender DNA damage sensitivity, HR defects, and cancer susceptibility to breast, ovarian, pancreatic whereas bi-allelic *PALB2* germline mutations cause Fanconi anemia subtype FANCN, with early onset of acute myeloid leukemia, medulloblastoma, neuroblastoma and often Wilms’ tumor [12–17]. Given the intimate functional links between *PALB2* and *BRCA2* and the similar phenotypes associated with biallelic mutations in the genes that encode them, it is plausible that monoallelic *PALB2* mutations confer susceptibility to adult cancer [12].

In Finland, Canada (in French-Canadians), and Poland, mutations in *PALB2* are the cause of between 0,5 and 1% of all breast cancers and 0,52% unselected cases of pancreatic cancer [13, 18–20]. We were interested to investigated if mutations in *PALB2* genes could be relevant to the pathogenesis of bladder or kidney cancer. Herein we genotyped 1413 patients with bladder and 810 cases with renal cancer and 4702 healthy controls**.**

## Material

### Patients

This study includes 1413 unselected cases of urothelial bladder cancer (376 women and 1037 men) and 810 unselected kidney cancer (360 women and 450 men) diagnosed at the Urology Hospital in Szczecin between 1986 and 2018. A total of 1518 incident cases of bladder cancer and 869 kidney cancer were identified during the study period. Of these, 1413 patients with bladder and 810 with kidney cancer accepted the invitation to participate (93, 93%). All patients had a histopathological diagnosis of cancer. All patients had a histopathological diagnosis of cancer. The mean age of diagnosis of bladder cancer patients was 68 years (range 13–91) and 62 (range 17–91) of kidney cancer. A family history was taken by the construction of family tree and the completion of a standardized questionnaire. A total of 45 patients with a family history of at least 1 bladder cancer in first or second degree relatives and 30 cases with a family history of at least 1 kidney cancer in first or second degree relatives were identified. Cigarette smoking was reported in 1045 (74%) cases with bladder and 488 (60%) kidney cancers. The vital status and the date of death of all of the cases were requested from the Polish Ministry of the Interior and Administration in February 2020, and were obtained in March 2020. In total we collected data of death of 729 (51%) patients with bladder and 204 (25%) kidney cancer. The study was approved by the Ethics Committee of Pomeranian Medical University in Szczecin.

### Controls

The control group included 4702 cancer-free, population-based, adults from (the genetically homogeneous population) of Poland. In order to estimate the frequency of the Polish founder mutations in the general population, fourth control groups were combined. The first control group were women age 24–84 years identified from the region of Szczecin. These controls are described in detail elsewhere [21]. The second control group consisted of 1717 cancer-free females aged 32–72 years who participated in mammography screening at eight different centers across Poland: Kielce, Legnica, Olsztyn, Poznan, Szczecin, Swidnica, Torun, and Zielona Góra and who provided a blood sample for DNA analysis. The third group of women included 1036 patients age 20–94 years selected at random from computerized lists of patients at family practices located in the region of Opole. And the last fourth group included 990 women age 50–66 years who participated in a colonoscopy screening programme for colorectal cancer in Szczecin, Bialystok, and Łódz. The allele frequencies for all variants in our control group were not dependent on age and the prevalence estimates of mutations in all genes were similar in younger and in older controls.

## Methods

DNA was isolated from 5 to 10 mL of peripheral blood. The two recurrent mutations of *PALB2* (509_510delGA and 172_175delTTGT) were genotyped as described previously [14, 22]. In brief, these variants were genotyped with a TaqMan assay (Life Technologies, Carlsbad, CA) using a LightCycler Real-Time PCR 480 System (Roche Life Science, Mannheim, Germany). Sanger direct sequencing was undertaken to confirm the presence of mutations, using a BigDye Terminator v3.1 Cycle Sequencing Kit (Life Technologies), according to the manufacturer’s protocol. In all reaction sets, positive and negative controls (without DNA) were used.

### Statistical analysis

#### Survival analysis

We followed up *PALB2* mutation carriers from the date of diagnosis until the date of death from any cause, or March 2020, if they were still alive. The median follow-up was 204 months. Due to a two variants of *PALB2* mutations (509_510delGA and 172_175delTTGT) were not statistical significant among bladder and kidney cancer patients we did not perform survival analysis.

#### Odds ratios

The prevalence of each of the two *PALB2* alleles was compared in cases and in controls, singly and in combination. Odds ratios were generated from two-by-two tables and statistical significance was assessed using the Fisher exact test where appropriate. The odds ratios were used as estimates of relative risk and additionally were adjusted for age, sex and pack-years of smoking by multiple logistic regression.

### Ethical statement

The study was performed in accordance with the principles of the Declaration of Helsinki. All patients and controls provided written informed consent.

## Results

### Bladder cancer

Bladder cancer cases and 4702 controls were successfully genotyped for the two *PALB2* variants. Among bladder cancer cases the *PALB2* mutations (two variants combined) were found in 0.35% of the patients and in 0.21% of the controls (OR = 1.7; 95% CI 0.56–4.88; *p* = 0.52) (Table 1). A *PALB2* mutation (509_510delGA) was present in four (0.3%) of 1413 cases with bladder cancer and in seven (0.15%) of 4702 controls (OR = 1.9; 95% CI 0.55–6.51; *p* = 0.5). A mutation 172_175delTTGT was detected in one patient with bladder cancer (6.55%) and three (0.06%) out of 4702 controls (OR = 1.1; 95% CI 0.11–10.7; *p* = 0.9). In the group of 1037 men we observed three (0.29%) mutations of 509_510delGA and among 376 womans were two (0.53%) mutations of *PALB2* gene one of each type. The information about smoking we collected from 1045 patients with bladder cancer including 123 (11.8%) nonsmokers and 922 (88.2%) smokers. We observed one *PALB2* mutation in person who did not smoke (0.8%) and four mutations among smokers (0.4%). The frequency of *PALB2* mutation was slights higher in non-smokers (OR = 1.9; 95% CI 0.21–17; *p* = 0.47). In 45 family cases with bladder cancer in first- and/or second-degree relatives we did not observed any of investigated mutations in gene *PALB*2. Three patients with bladder cancer and mutation in variant 509_510delGA died up to a year after diagnosis and one to March 2020 was still alive. Patient with mutation in 172_175delTTGT died half year after diagnosis of the bladder cancer.

**Table 1:** Effect of *PALB2* Mutations on Bladder Cancer Risk

Mutation subjects	Number of carriers/total (frequency %)	OR	95% CI	***p***-value
509_510delGA				
Controls	7/4702 (0.15)	1.0		
Cases	4/1413 (0.3)	1.9	0.55–6.51	0.5
172_175delTTGT				
Controls	3/4702 (0.06)	1.0		
Cases	1/1413 (6.55)	1.1	0.11–10.7	0.9
Any *PALB2* mutation				
Controls	10/4702 (0.21)	1.0		
Cases	5/1413 (0.35)	1.7	0.56–4.88	0.52

### Kidney cancer

The 810 kidney cancer cases and 4702 controls were successfully genotyped for the two *PALB2* variants. In kidney cases *PALB2* mutations (two variants combined) were found in 0.24% of the patients and in 0.21% of the controls (OR = 1.2; 95% CI 0.25–5.13; *p* = 0.84) (Table 2). The *PALB2* mutations (509_510delGA) were present in one (0.1%) of 810 cases with kidney cancer and in seven (0.15%) of 4702 controls (OR = 0.8; 95% CI, 0.10–6.75; *p* = 0.86). A mutation 172_175delTTGT was detected in one patient with kidney cancer (0.1%) and three (0.06%) out of 4702 controls (OR = 1.9; 95% CI, 0.20–18.6; *p* = 0.56). In the group of 450 men we observed two (0.45%) mutations of PALB2 gene one of each type. The information about smoking we collected from 488 patients with kidney cancer including 190 (39%) nonsmokers and 298 (61%) smokers. The one mutation of variant 509_510delGA was observed in person who smoked less than 20 of pack years (0.33%). We did not have information about smoking in patients with mutation 172_175delTTGT in *PALB2* gene. In 30 family cases with kidney cancer in first- and/or second-degree relatives we did not observed any of investigated mutations in gene *PALB*2. The patient with kidney cancer and mutation in 172_175delTTGT died 3 years after kidney cancer of diagnosis but the patient with mutation in second investigated variant of gene *PALB2* was still alive until March 2020.

**Table 2:** Effect of *PALB2* Mutations on Kidney Cancer Risk

Mutation subjects	Number of carriers/total (frequency %)	OR	95% CI	***p***-value
509_510delGA				
Controls	7/4702 (0.15)	1.0		
Cases	1/810 (0.1)	0.8	0.10–6.75	0.86
172_175delTTGT				
Controls	3/4702 (0.06)	1.0		
Cases	1/810 (0.1)	1.9	0.20–18.6	0.56
Any *PALB2* mutation				
Controls	10/4702 (0.21)	1.0		
Cases	2/810 (0.24)	1.2	0.25–5.13	0.84

## Discussion

The results of our unselected cohort 1413 bladder, 810 kidney cancer cases and 4702 controls revealed no statistical significant, indicating that two mutations of *PALB2* gene (509_510delGA and 172_175delTTGT) do not seem to play a major role in bladder or kidney cancer development. The *PALB2* mutations combined are rare in the general population (0.21%). In this study we found that mutations in *PALB2* gene were seen in five (0.35%) unselected cases of bladder cancer and two (0.24%) unselected cases of kidney cancer. Due to low statistical power of the study 18.6% for bladder cancer and 5.2% for kidney cancer our results need to be confirmed by larger multi-center study.

In the literature there are some studies of *PALB2* in unselected bladder and kidney cancer cases but again they are based upon small study cohorts. Reid et al. have described bi-allelic mutations in *PALB2* in seven families affected with Fanconi anemia and cancer in early childhood [12]. Although *PALB2* mutations were less common overall and appreciated in only 0.6% of tumors tested a significant proportion of *PALB2* mutations were found in bladder (1.49%), breast (1.05%) but no single mutation was found in kidney. Adank et al screened a random cohort of 47 Dutch Wilms tumor patients for germline mutations in *PALB2* by DNA sequencing and Multiplex Ligation-dependent Probe Amplification and they did not identify any bi-allelic pathogenic mutations [23]. Heeke et al. tested 201 bladder and 199 kidney tumors. Thy found that frequency of gene *PALB2* mutation was 1.49% in bladder cancer and 0% in kidney cancer tumors [24]. Lee Yap et al. found five somatic mutations of *PALB2* gene in two cases of bladder cancer [25]. Lee Yap et al. also observed that in patients with mutations in DNA repair genes is longer recurrence-free survival. In this study we did not do multivariable analysis because the presence of a *PALB2* (509_510delGA and 172_175delTTGT) mutations were not statistical significant among bladder and kidney cancer patients.

In summary we found no difference in the prevalence of recurrent *PALB2* mutations between cases and healthy controls. Our results indicate that testing mutations 509_510delGA and 172_175delTTGT is unlikely to be relevant for the identification of individuals at risk of bladder or kidney cancer, at least in the Polish population.

## Data Availability

Availability of data and materials are include in manuscripts.
